# Animal models of attention-deficit hyperactivity disorder

**DOI:** 10.1186/1744-9081-1-9

**Published:** 2005-07-15

**Authors:** Vivienne A Russell, Terje Sagvolden, Espen Borgå Johansen

**Affiliations:** 1 Center for Advanced Study at the Norwegian Academy of Science and Letters, Oslo, Norway; 2 Department of Human Biology, University of Cape Town, South Africa; 3 Department of Physiology, Institute of Basic Medical Sciences, University of Oslo, Norway

## Abstract

Although animals cannot be used to study complex human behaviour such as language, they do have similar basic functions. In fact, human disorders that have animal models are better understood than disorders that do not. ADHD is a heterogeneous disorder. The relatively simple nervous systems of rodent models have enabled identification of neurobiological changes that underlie certain aspects of ADHD behaviour. Several animal models of ADHD suggest that the dopaminergic system is functionally impaired. Some animal models have decreased extracellular dopamine concentrations and upregulated postsynaptic dopamine D1 receptors (DRD1) while others have increased extracellular dopamine concentrations. In the latter case, dopamine pathways are suggested to be hyperactive. However, stimulus-evoked release of dopamine is often decreased in these models, which is consistent with impaired dopamine transmission. It is possible that the behavioural characteristics of ADHD result from impaired dopamine modulation of neurotransmission in cortico-striato-thalamo-cortical circuits. There is considerable evidence to suggest that the noradrenergic system is poorly controlled by hypofunctional α_2_-autoreceptors in some models, giving rise to inappropriately increased release of norepinephrine. Aspects of ADHD behaviour may result from an imbalance between increased noradrenergic and decreased dopaminergic regulation of neural circuits that involve the prefrontal cortex. Animal models of ADHD also suggest that neural circuits may be altered in the brains of children with ADHD. It is therefore of particular importance to study animal models of the disorder and not normal animals. Evidence obtained from animal models suggests that psychostimulants may not be acting on the dopamine transporter to produce the expected increase in extracellular dopamine concentration in ADHD. There is evidence to suggest that psychostimulants may decrease motor activity by increasing serotonin levels. In addition to providing unique insights into the neurobiology of ADHD, animal models are also being used to test new drugs that can be used to alleviate the symptoms of ADHD.

## Introduction

Attention-deficit/hyperactivity disorder (ADHD) is the most commonly diagnosed psychiatric disorder of childhood [1,2]. Children with ADHD are characterized by an inability to sit still, they have difficulty organizing tasks, they are forgetful, have a tendency to be easily distracted, fidget, they have difficulty with tasks that require sustained attention and are risk-takers [3,4]. Their behaviour falls into two or three core clusters of symptoms, impaired sustained attention and hyperactivity/impulsiveness that develops gradually in familiar situations, with impairment manifesting before age 7 [3,5].

The high population prevalence and heritability of ADHD agrees with ADHD being caused by multiple genes with small effect size [2,6]. Associations have been found between polymorphisms in several monoaminergic genes and ADHD. These include the dopamine D1, D4 and D5 receptor (DRD1, DRD4, DRD5) genes, the α_2_-adrenoceptor gene, dopamine, norepinephrine and serotonin transporter (DAT1, NET1, SERT1) genes [7-19]. Contradictory negative findings have also been reported suggesting that different combinations of genetic factors may combine to produce individual clusters of behavioural characteristics of ADHD [20-23]. Different alleles of genes encoding proteins related to dopamine function differentially affect cognitive function [24]. The effect of a single gene on behaviour has been described as small causing a slight bias towards one end of a continuum [4,24].

Numerous studies have found reduced brain volume in ADHD patients, particularly prefrontal cortex, cerebellum, corpus callosum, and basal ganglia [25-29]. Dopamine alters brain structure and function [24]. The DAT1 genotype preferentially influenced caudate volume; individuals homozygous for the 10-repeat allele which is associated with ADHD had smaller caudate volumes than individuals carrying the 9-repeat allele [24]. The DRD4 genotype influenced prefrontal gray matter. Individuals homozygous for the 4-repeat allele had smaller volumes than individuals carrying other variants of the gene [24]. Imaging studies have demonstrated functional abnormalities in striatum, frontal cortex and cerebellum of patients with ADHD [30-34].

Imaging studies have revealed robust increases in striatal DAT of up to 70% in ADHD children and adults [10,15,35]. Although not every study found increased DAT [36,37], there is a strong possibility that the DAT1 gene is overexpressed in the striatum of ADHD subjects, and that this results in reduced synaptic dopamine.

Psychostimulants are highly effective in ameliorating the three major clusters of behavioural symptoms of ADHD [38]. Methylphenidate produced improvements in spatial working memory, attentional set-shifting [39,40], and inhibition of previously acquired behavioural responses to non-relevant stimuli [34,41-43]. It increased the previously reduced striatal activity in patients with ADHD [34] and reduced cerebral blood flow in frontal and parietal cortex [39,44].

### Animal models of ADHD

Although non-human primate brains are closer to human brains than rodents, rodent models of ADHD have the advantage that they are genetically more homogeneous, they are less expensive to maintain, greater numbers of experimental animals are available so they are not used for multiple studies, and much more is known about their neurobiology than primates. The researcher also has better control over variables such as diet, environment, and learning history. Rodent models have simpler nervous systems, they cannot be used to study complex cognitive behaviour like language but their basic behavioural mechanisms are similar to humans.

ADHD is a heterogeneous disorder with individual patients presenting with quite different behavioural symptoms probably as a result of different combinations of genetic and environmental factors. Animal models provide invaluable insight into the neurochemistry underlying specific aspects of ADHD behaviour, when compared to appropriate controls. Differences between the behaviour of an animal model and its control can be correlated with differences in their neurochemistry and behavioural pharmacology.

A list of criteria for an optimal animal model of ADHD was recently suggested [45] (i) the model should mimic the fundamental behavioural characteristics of ADHD (face validity), impulsiveness should be absent initially and develop gradually over time, sustained attention-deficit should be demonstrated only when stimuli are widely spaced in time, hyperactivity should not be observed in a novel, non-threatening environment, it should develop over time; (ii) the model should conform to a theoretical rationale for ADHD (construct validity): the two main behavioural processes that are proposed to be major contributory factors in the aetiology of ADHD, altered reinforcement of novel behaviour and deficient extinction of previously reinforced behaviour, should be demonstrated; (iii) the model should predict novel aspects of ADHD behaviour, genetics, and neurobiology (predictive validity); and (iv) it should be neurodevelopmental, preferably a genetic model.

We use the concept 'reinforcer' strictly in a behavioural sense, without making any references to subjective or cognitive states. The alternative concept of 'reward' is more cognitive and may connote several subjective states like pleasure as well as incentive and reinforcer [46]. Therefore, there is not a perfect overlap between reinforcer and reward. We prefer the more descriptive and less ambiguously defined concept of reinforcer rather than reward [4].

Spontaneously hypertensive rats (SHR) were found to be the best characterized and also currently the most appropriate model of ADHD [45]. SHR fulfil most of the validation criteria listed above and compare well with clinical cases of ADHD [45,47]. Poor performers in the 5-choice serial reaction time (5-CSRT) task were suggested to be a useful model for the inattentive subtype of ADHD [45]. Other animal models were suggested to provide useful information concerning aspects of ADHD behaviour [45].

### SHR

SHR exhibit all the behavioural characteristics of ADHD: impaired sustained attention without obvious sensory problems, motor impulsiveness, and hyperactivity that is not present in novel, non-threatening situations but develops over time when reinforcers are infrequent [45,47]. Similar to children with ADHD, SHR display increased behavioural variability, deficient response re-engagement, and make significantly more errors than controls [45,47,48].

Besides conforming to behavioural criteria for an animal model of ADHD, SHR fulfils the additional criterion that it is a genetic model of ADHD bred from progenitor Wistar Kyoto (WKY) rats [49]. WKY serve as a valid control for SHR since their behavioural characteristics are similar to those of other rat strains [47]. Three candidate dopamine genes (DRD2, DRD4, and DAT) were sequenced in SHR and WKY [Mill et al., unpublished]. No differences were found in DRD2 or DRD4 genes but a 160 bp insertion was found in the non-coding region upstream of exon 3 of the DAT1 gene which is of significance since variable number tandem repeats in the 3'-untranslated region of the DAT gene has been associated with ADHD in several family studies [7,9,10,14,15]. A possible disturbance in the regulation of the DAT1 gene is in agreement with findings that DAT1 gene expression is transiently reduced in SHR midbrain during the first postnatal month and increased in adult SHR compared to controls [50,51]. Alterations in DAT1 gene expression can affect dopamine uptake and reutilization. Decreased expression of DAT1 will reduce reuptake and increase metabolism of dopamine. Differences in dopamine metabolism have been reported for children and adults with ADHD [52,53] which is consistent with developmental changes in DAT1 expression and consequent changes in dopamine uptake. DOPA decarboxylase activity was found to be increased in the midbrain of children and decreased in prefrontal cortex of adults with ADHD compared to controls [52,53]. Reduced DAT1 expression at a young age would reduce dopamine reuptake, thereby reducing dopamine reutilization and necessitating increased synthesis of dopamine by DOPA decarboxylase. In adults, increased expression of DAT1 might be expected to increase reuptake of dopamine, thereby reducing the need for synthesis by DOPA decarboxylase.

SHR appear to have higher extracellular tonic dopamine in the nucleus accumbens shell [54]. However, consistent with increased DAT1 expression in adult SHR striatum, extracellular dopamine levels are decreased in the caudate nucleus [55,56] and d-amphetamine-stimulated release of dopamine via DAT1 is greater in SHR striatum than WKY [54,57,58]. Evidence suggests that DAT1 is hypofunctional in SHR, since despite the increased number of DATs, inhibition of dopamine uptake by low concentrations of methylphenidate or nomifensine increased the electrically-stimulated release of dopamine to the same extent in SHR and WKY nucleus accumbens and caudate-putamen [58,59]. These findings suggest that increased expression of the DAT1 gene may reflect an attempt to compensate for increased tonic extracellular dopamine in the nucleus accumbens shell of SHR or increased DAT1 expression may occur in an attempt to compensate for decreased function of DAT1 in adult SHR striatum.

Hypertension is a confounding factor in the SHR model of ADHD. However, SHR do not develop hypertension until they are adults, from 10 to 12 weeks of age, whereas hyperactivity is observed at 3 to 4 weeks of age before they enter puberty [55]. In an attempt to map quantitative trait loci for complex phenotypes, SHR were crossed with a Brown Norway congenic strain to generate a set of recombinant inbred strains [60]. Analysis of their behaviour revealed that locomotion mapped to chromosomes 3, 8 and 18 while hypertension exhibited multigenic complexity with both environment and genetic background as contributing factors. Elevated arterial blood pressure was higher when measured by direct catheterization compared to radiotelemetry suggesting that SHR hypertension is a product of stress-dependent trait expression [60]. SHR behaviour was suggested to result from an interaction between genetics and the environment [60], much like ADHD [4,6,23].

In addition to behavioural and genetic similarities to ADHD, SHR exhibit brain pathology similar to ADHD. SHR brain volumes, specifically prefrontal cortex, occipital cortex, and hippocampus, are smaller than controls [61]. MRI revealed significantly increased ventricular volume in SHR compared to WKY at 3 months of age [61]. There are fewer neurons in these brain areas compared to WKY [62-64].

Results obtained with SHR have predicted novel alternatives to existing theories concerning the aetiology of ADHD. A major second messenger system involving calcium signalling is dysfunctional in SHR [65,66] suggesting that several neurotransmitter systems could be impaired in ADHD. Compared to WKY, SHR have lower brain Ca^2+ ^ATPase activity [67]. Because neurotransmitter release is dependent on calcium influx, a disturbance in the concentration gradient of calcium across the cell membrane may decrease the influx of calcium ions into the cell and impair neurotransmitter release [65]. Decreased calcium influx through NMDA channels would also impair intra- and intercellular signalling as well as LTP, the neuronal analogue of learning [4,68].

Calcyon, a transmembrane protein involved in DRD1/DRD5 signalling, has been implicated in ADHD [69]. Polymorphisms of the calcyon gene (DRD1IP) have been associated with both inattentive and hyperactive/impulsive subtypes of ADHD [Laurin et al., submitted]. Calcyon enables the typically Gs-linked DRD1/DRD5 to switch from Gs to Gq coupling, thereby stimulating inositol 1,4,5-triphosphate (IP3) turnover with resultant release of calcium from intracellular stores. This effect requires a priming step involving heterologous Gq-linked G-protein-coupled receptor activation. Subsequent DRD1 activation causes elevation of intracellular calcium that triggers calcyon accumulation in the plasma membrane [70]. SHR appear to have a disturbance in calcium metabolism. Increased intracellular calcium could interfere with calcyon translocation to the cell membrane and thereby impair DRD1/DRD5 function. Alternatively, altered calcyon function could impair DRD1/DRD5 function by affecting the switch from Gs to Gq coupling, thereby also altering intracellular calcium concentration. These findings suggest that the primary disturbance in ADHD may be located in factors that regulate postsynaptic DRD1/DRD5 signalling mechanisms, hence, calcyon and other DRD1/DRD5 signalling-related proteins/peptides deserve further investigation [Laurin et al, submitted]. The hypothesis that calcyon may be a primary disturbance in ADHD could be tested in animal models, particularly SHR.

#### Dopamine

Dopamine neurons play an important modulatory role in the brain [71]. Neurons that release dopamine influence behaviour by exerting modulatory effects on the transfer of information through neuronal circuits that connect functionally distinct cortical areas to specific regions of the striatum in parallel cortico-striato-thalamo-cortical pathways. Dopamine assists in reprogramming the brain by selectively reinforcing the weights of the synapses that are active around the time of behavioural reinforcement [4,72].

There are three major dopaminergic systems in the brain, the mesolimbic, mesocortical and nigrostriatal pathways. Mesolimbic dopamine neurons project from the ventral tegmental area of the midbrain (VTA) to limbic areas of the brain. The firing rate of dopamine neurons is increased in response to unexpected reward and decreased when a fully predicted reward is omitted [73,74]. It has been suggested that deficient reinforcement of appropriate behaviour and/or deficient extinction of previously reinforced behaviour can give rise to ADHD symptoms of delay aversion, hyperactivity in a familiar environment, impulsiveness, deficient sustained attention, increased behavioural variability and failure to extinguish previously acquired behaviour [4].

The mesocortical dopamine system originates in the VTA and projects to cortical areas, including the prefrontal, parietal and temporal cortex. These dopamine projections modulate circuits that are known to play an important role in a variety of executive functions, including motor control, behavioural inhibition, attention, and working memory [4,75]. Dopamine activation of DRD2 selectively modulates neural activities associated with memory-guided motor activity in delayed response tasks whereas DRD1 are responsible for memory-related persistent activation of prefrontal cortex neurons [76]. Deficient dopamine-mediated modulation of prefrontal cortical circuits has been suggested [4] to cause attention response deficiencies (impaired orienting responses, saccadic eye movements and responses towards a target) and impaired executive functions (poor behavioural planning).

Nigrostriatal dopamine neurons project from the substantia nigra pars compacta to the dorsal striatum (caudate nucleus and putamen). Impaired dopamine modulation of cortico-striato-thalamo-cortical circuits can impair motor function and cause deficient habit learning i.e. impaired nondeclarative memory formation [4]. These impairments can give rise to apparent developmental delay, clumsiness, and neurological "soft signs" [4].

Dopamine uptake, storage and/or metabolism are disturbed in SHR [58,77], as has been proposed for children and adults with ADHD [52,53]. Although the tissue concentration of dopamine, which reflects vesicle stores of dopamine, is similar in SHR and WKY [78], dopamine turnover which reflects release and metabolism of dopamine was lower in adult SHR substantia nigra, VTA, striatum and frontal cortex [78,79]. The dopamine metabolite, homovanillic acid, and the homovanillic acid /dopamine ratio are much lower in several brain areas of adult SHR compared to WKY. These findings are consistent with increased expression of DAT1 increasing dopamine re-uptake and reutilization thereby reducing metabolism. The results could also be interpreted to suggest that dopamine release is decreased and dopamine transmission is impaired in SHR.

*In vitro *stimulation-evoked (electrical and/or exposure to high K^+ ^concentration) release of dopamine from terminals of mesocortical, mesolimbic and nigrostriatal dopamine neurons of SHR is significantly less than that of WKY [55,56,58,59,77,80]. A similar decrease in stimulus-evoked release of dopamine was observed in SHR nucleus accumbens shell *in vivo *[54]. The elevated dopamine concentration in the nucleus accumbens shell may have increased activation of endogenous DRD2 autoreceptors and thus reduced stimulus-evoked dopamine release [54]. *In vitro *DRD2-mediated inhibition of dopamine release was greater in SHR caudate-putamen and nucleus accumbens than WKY, while DRD2 function was unchanged in prefrontal cortex of SHR [77]. Results are consistent with increased endogenous activation of DRD2 in the nucleus accumbens while increased efficacy of endogenous dopamine activation of DRD2 autoreceptors was suggested to account for the decreased stimulus-evoked release of dopamine in SHR striatum [77].

Increased DRD2 function was suggested to have occurred in a compensatory reaction to abnormally elevated dopamine levels at an early stage of development, perhaps as a result of exposure to stress and/or a genetic defect [80]. A likely explanation could also be that elevated extracellular dopamine levels could have resulted from decreased DAT1 expression during the first postnatal month of SHR [50] which caused compensatory upregulation of both DAT1 and DRD2 receptors [51,58,77]. Increased d-amphetamine stimulated, DAT-mediated, release of dopamine from SHR prefrontal cortex and striatum is consistent with upregulation of DAT1 in adult SHR [58].

In agreement with decreased stimulus-evoked release of dopamine, postsynaptic DRD1 are increased in the caudate-putamen and nucleus accumbens of SHR [51,81,82] suggesting compensatory upregulation of postsynaptic receptors.

Dopamine dysfunction may contribute to the altered reinforcement processes of ADHD [4,45]. DRD1 mediates reinforcement by strengthening synaptic connections between neurons (long-term potentiation, LTP) or weakening synaptic connections (long-term depression, LTD) in neural circuits that involve prefrontal cortex and/or striatum (e.g. cortico-striato-thalamo-cortical circuits) [74,83,84]. LTP is regarded as a neuronal correlate of learning [68]. It requires interplay between several factors. Among these is coincident glutamate stimulation of NMDA receptors and local membrane depolarization sufficient to remove the magnesium block of NMDA receptor channels. Glutamate activation of AMPA receptors allows influx of sodium ions and can thereby produce this depolarization. Activation of the DRD1-protein kinase A signalling pathway increases the mobilisation of AMPA and NMDA receptors to the cell surface, thereby promoting LTP [85-87].

Calcium enters the cell through the NMDA receptor channel, activates various protein kinases including calcium/calmodulin-dependent protein kinase II, and mobilizes "silent" AMPA receptors required for LTP to take place [68]. NMDA receptor-induced excitation is enhanced by DRD1 activation and attenuated by DRD2 activation [88-90]. Thus DRD1 activation may synergistically increase the excitatory actions of glutamate at NMDA receptors, increasing the open time of NMDA channels and therefore increase the calcium signal. Activation of DRD1 receptors is required for stimulation of cAMP formation, and subsequent activation of cAMP-dependent protein kinase, phosphorylation of CREB and gene transcription required for consolidation of memory traces [91].

NMDA receptors are required for LTP in the hippocampus [68], in cortico-striatal synapses [92], and in the nucleus accumbens [93]. Phasic application of dopamine in the prefrontal cortex facilitates LTP, suggesting that dopamine can promote reinforcement processes by strengthening (or weakening) network connections in the prefrontal cortex [94]. Bilateral infusion of a DRD1 agonist increased attentional accuracy and facilitated short-term spatial memory after delays of several seconds and impaired memory of the location of a visual target after short delays, thereby modulating short-term working memory in a delay-dependent manner [95]. Effective decision making requires the ability to adapt behaviour on the basis of changes in emotional significance. Rats with lesions of the orbitofrontal cortex showed increased preference for larger but delayed rewards whereas rats with lesions of the basolateral amygdala showed increased choice of small immediate rewards [96].

Within the striatum, LTP (and LTD) only occurs in the presence of dopaminergic input [97]. Consistent with decreased DRD1 activation in SHR giving rise to decreased facilitation of NMDA receptor function and impaired gene transcription, SHR have reduced expression of calcium/calmodulin-dependent protein kinase II and c-fos gene in the anterior striatum [98-100]. Decreased DRD1 function will alter transmission of signals in cortical and striatal circuits of SHR and impair dopamine-mediated cognitive function and reinforcement of appropriate behaviour.

Deficient DRD1 function in SHR implies that only stimuli with strong reinforcer value will release enough DA to stimulate DRD1 sufficiently to facilitate NMDA receptor function and produce phosphorylation of CREB and other important proteins/peptides required for strengthening of synaptic connections in circuits representing goal-directed learning and memory consolidation. Weak stimuli will not cause behaviour to be reinforced in SHR because of reduced activation of the DRD1 signalling pathways. As suggested in the dynamic developmental behavioural theory (DDT) of Sagvolden et al [4], SHR have a steeper delay gradient, so decreased activation of DRD1 signalling pathways in response to an unexpected reinforcer would not strengthen synaptic connections in circuits that were activated by stimuli some time prior to the reinforcer with the result that more recently activated behavioural circuits would be preferentially strengthened and their memory consolidated.

The striatum is central to behavioural control and receives the greatest density of dopamine input of all central nervous structures. Striatal dopamine hypofunction may be associated with subtle motor control problems in children with ADHD and SHR [4]. Impaired dopamine regulation of striatal function may contribute to the poor motor development associated with severe cases of ADHD [101]. Response "disinhibition" may be due to impaired motor control associated with dopamine hypofunction in the striatum rather than frontal lobe dysfunction [4]. Indeed, motor control problems may explain a number of effects including clumsiness, increased variability in speed, less accurate response re-engagement, "failure to inhibit" responses when quick reactions are required, impaired orienting responses, increased number of responses with extended reaction times, apparent developmental delay, neurological "soft signs", and language delays [4].

Papa et al. [100] found decreased calcium/calmodulin-dependent protein kinase II in the nucleus accumbens shell but not the core of SHR when compared to WKY. The mesolimbic dopamine projection to the shell subdivision of the nucleus accumbens is responsible for motivation and determines the amount of effort an animal is prepared to exert in order to obtain a reward. Hypofunction of the mesolimbic dopamine system will impair the function of the mesocortical and nigrostriatal dopamine systems by influencing dopamine release and the cortico-striato-thalamo-cortical circuits that dopamine modulates. This could impair learning and expression of goal-directed behaviour thereby contributing to the aspects of ADHD symptoms displayed by SHR [102].

#### Norepinephrine

In addition to the hypothesis that dopaminergic systems are hypofunctional in ADHD, noradrenergic neurons have been suggested to be poorly regulated and hyperfunctional in the prefrontal cortex of children with ADHD [40,103,104]. Noradrenergic neurons appear to enhance the signal-to-noise ratio in prefrontal and parietal cortices, amplify responses to attended stimuli, and reduce responses to irrelevant stimuli [71,105]. Both of these functions are defective in ADHD [71].

The locus coeruleus diffusely innervates diverse regions throughout the central nervous system including the entire cerebral cortex, various subcortical areas, cerebellum and spinal cord, and plays an important role in attention, arousal, orienting, and vigilance [40]. For example, locus coeruleus neurons respond selectively to attended (target) stimuli; tonic locus coeruleus activity corresponds to arousal state, and both very low and very high locus coeruleus activity are associated with impaired vigilance [103,105]. Noradrenergic neurons that project from the locus coeruleus to the prefrontal cortex release norepinephrine which helps to guide behaviour by modulating the transfer of information through neuronal circuits that are responsible for selective and sustained attention [40].

Norepinephrine, like dopamine, alters the strength of neural connections leading to adaptive changes in behaviour. This occurs through activation of β-adrenoceptors that stimulate cAMP formation, with subsequent activation of cAMP-dependent protein kinase, phosphorylation of CREB and gene transcription required for consolidation of memory traces in several brain areas, including amygdala and hippocampus [91,106]. Dopamine and norepinephrine act in concert to regulate prefrontal cortex function and thereby ensure appropriate behaviour [103]. α_2_-Adrenoceptors agonists enhance performance of tasks requiring prefrontal cortex function while α_1_-adrenoceptor agonists impair prefrontal cortex function [103]. Furthermore, α_1_- and α_2_-antagonists reverse these effects [103,107]. Either excessive activation of α_1_-adrenoceptors or deficient α_2_-adrenoceptor mediated modulation of prefrontal cortical circuits can impair prefrontal cortex function [103,108]. The prefrontal cortex projects to the VTA and locus coeruleus, thereby influencing the firing rate of both dopamine and norepinephrine neurons and impacting on many cognitive processes [103].

The highly specific antagonist of NET1, atomoxetine, is as effective as methylphenidate in treating ADHD [38,109], further emphasizing an important role for the noradrenergic system in the disorder. However, atomoxetine also increases synaptic availability of dopamine in the prefrontal cortex [110] which may contribute to its beneficial effects. Drugs used to treat ADHD symptoms are likely to have different effects on different neurotransmitter systems. Drugs that act on the noradrenergic system, such as atomoxetine, tricyclic antidepressants like the NET1 blocker, desipramine, and α_2_-adrenoceptor agonists such as clonidine and guanfacine, have a different therapeutic time-course compared to psychostimulants. Methylphenidate produces amelioration of ADHD symptoms within 30 minutes and is short-acting whereas noradrenergic drugs have to be administered for longer periods of time before a therapeutic effect is observed, and improvement is sustained for several months [38]. This suggests that noradrenergic drugs cause long-term adaptive changes that are therapeutic. Chronic treatment with desipramine produces a series of changes in presynaptic and postsynaptic α- and β-adrenoceptors and causes long-term downregulation of cortical β-adrenoceptors [111-113]. This suggests that behavioural improvement can perhaps be achieved by decreased noradrenergic activation of cortical β-adrenoceptors, thereby decreasing noradrenergic function, which is consistent with a hyperactive noradrenergic system and the dopamine/norepinephrine imbalance hypothesis of ADHD.

Disturbances in norepinephrine metabolism in SHR are suggested by the finding that tyrosine hydroxylase gene expression is higher in the ventrolateral medulla oblongata of SHR than WKY [114], consistent with elevated norepinephrine concentration in several brain areas of SHR including locus coeruleus, substantia nigra and prefrontal cortex [79]. Increased norepinephrine concentrations in SHR brain is consistent with downregulation of β-adrenoceptors in cerebral cortex of SHR [115]. Furthermore, NET1 function is increased in SHR cerebral cortex [115] which could increase uptake of dopamine into noradrenergic terminals and varicosities and deplete extracellular dopamine in the prefrontal cortex [116].

Evidence suggests that there is an imbalance between dopaminergic and noradrenergic neurotransmission in the prefrontal cortex of SHR [104]. While dopamine release is decreased in SHR prefrontal cortex, norepinephrine concentrations are elevated. The noradrenergic system appears to be hyperactive as a result of impaired α_2_-autoreceptor regulation [104].

Stimulus-evoked (electrically stimulated or K^+^-evoked) release of norepinephrine from prefrontal cortex slices of SHR was no different from that of WKY [117]. However, α_2A_-adrenoceptor mRNA levels were decreased in SHR compared to WKY and α_2_-autoreceptor-mediated inhibition of norepinephrine release was less efficient in SHR than in WKY suggesting that α_2_-adrenoceptor function is impaired [117-119]. α_2A_-Adrenoceptors are the subtype specifically expressed in the prefrontal cortex, so impaired α_2A_-adrenoceptor function would be expected to impair cognition [103,120].

Decreased α_2_-autoreceptor-mediated regulation of norepinephrine neurons and impaired inhibition of norepinephrine release may be particularly disruptive to the function of target structures when the firing rate of locus coeruleus neurons is high, causing excessive spillover of norepinephrine. Repeatedly increased release of norepinephrine from sympathetic nerve terminals could give rise to the stress-dependent [60] development of hypertension in SHR. Expression of the gene encoding G_iα _the G-protein subunit that inhibits cAMP formation from ATP by adenylyl cyclase is increased in SHR aorta at 2 weeks of age, possibly reflecting an attempt by a target organ to decrease the effect of increased norepinephrine release from sympathetic nerve endings. Poorly controlled norepinephrine release could also give rise to excessive activation of α_1_-adrenoceptors in the prefrontal cortex impairing its function. Other noradrenergic terminal areas in the central nervous system may be similarly affected. These findings suggest that the noradrenergic system is hyperactive in SHR, particularly in response to stress, and supports the hypothesis that there is an imbalance between norepinephrine hyperfunction and dopamine hypofunction in ADHD.

#### Serotonin

Brain serotonin (5-hydroxytryptamine, 5-HT) function has been suggested to be altered in SHR [121]. Higher serum testosterone, and lower amygdala serotonin content has been associated with a mutation in the non-pseudoautosomal region unique to the Y-chromosome of SHR [122]. Administration of a serotonin transporter inhibitor, fenfluramine, evoked less prolactin secretion in SHR than WKY [121]. Acute administration of the selective serotonin reuptake inhibitor, citalopram, reduced hyperactivity of SHR in an elevated plus-maze [123]. However, there was no difference between SHR and WKY in midbrain, hippocampal, or striatal serotonin concentration or serotonin uptake kinetics [123]. In addition, stressors released serotonin in the locus coeruleus of SHR and WKY rats to the same extent [124] and 5-HT_2C _receptor function was reported to be unaltered in SHR compared to WKY [125]. These findings do not support a role for serotonin in the aetiology of ADHD symptoms in SHR.

#### Glutamate

In addition to decreased autoreceptor-mediated inhibition of norepinephrine release from SHR prefrontal cortex slices, glutamate activation of AMPA receptors caused greater release of norepinephrine from SHR prefrontal cortex slices than WKY [126,127]. Glutamate is present in micromolar concentrations in the extracellular space outside the synaptic cleft and regulates tonic dopamine concentration in the extracellular fluid [128-132]. Dopamine release is increased by activation of AMPA receptors in rat striatum [133,134]. Glutamate activation of NMDA receptors upregulates DRD1 function by a direct protein-protein interaction at the carboxy terminals of both receptors [135]. As suggested by Seeman and Madras [136], the common defect in ADHD could be decreased extracellular dopamine levels. This deficiency could result from increased expression of DAT1, impaired dopamine synthesis, impaired release, or impaired regulation of extracellular dopamine by glutamate afferents from the prefrontal cortex, hippocampus, or amygdala [102]. However, *in vitro *activation of AMPA receptors caused similar fractional release of dopamine from SHR and WKY nucleus accumbens core [134]. Unlike WKY, glutamate-stimulated release of dopamine from the shell subdivision of SHR nucleus accumbens was significantly lower than from the core subdivision of SHR, suggesting that the shell may be particularly vulnerable to disturbances in dopamine release [134].

Neural circuits that use glutamate as a neurotransmitter are modulated by dopamine and norepinephrine. Future studies should be aimed at investigating glutamate function in the brains of SHR. In particular, measuring glutamate release in the prefrontal cortex and nucleus accumbens using *in vivo *microdialysis during reinforcement and extinction (hypothesized to be the major causative factors underlying ADHD [4]) may provide useful information concerning afferent glutamate input to these brain areas as a result of the additional demands of such tasks.

#### Psychostimulants

Psychostimulants are the most effective drugs used in the treatment of ADHD and provide a powerful means to gain insight into the underlying disturbances of ADHD. d-Amphetamine and methylphenidate reduced the ADHD-like behaviour of SHR [137] [Sagvolden, unpublished; Russell, unpublished]. The increase in DRD1 density observed in SHR striatum is reversed by methylphenidate treatment suggesting that psychostimulants reduce ADHD-like behaviour of SHR by increasing dopamine activation of DRD1 [51,81,82] thereby enabling dopamine-mediated LTP and reinforcement mechanisms to take place.

Psychostimulants changed the performance of SHR in fixed-interval schedules of reinforcement of bar-presses by lengthening the delay-of-reinforcement gradient. However, WKY performance changed to a greater extent than SHR, suggesting that the effect of psychostimulant drugs was less pronounced in SHR than in WKY [138]. The reduced reactivity to psychostimulants may be associated with abnormalities in DAT1 gene expression [50,51] [Mill et al., unpublished] and dopamine hypofunction as a result of adaptation to increased availability of dopamine at an early stage of development with subsequent reorganization of neural mechanisms that control VTA dopamine neuron function [139]. Further support for the hypothesis that regulation of midbrain dopamine neurons is altered in SHR was provided by the fact that repeated administration of methylphenidate (2.5 mg/kg) elicited locomotor sensitization i.e. increased locomotor response to the same dose of methylphenidate 3 days after cessation of treatment in Sprague-Dawley and WKY rats but not in SHR who were unaffected by the drug [140]. *In vitro *findings provided further support, where methylphenidate released significantly less dopamine from SHR nucleus accumbens slices than WKY [58], and chronic methylphenidate treatment (3 mg/kg for 2 weeks) increased endogenous dopamine activation of DRD2 in WKY striatum but did not alter DRD2 function in SHR probably because DRD2 were already up-regulated in SHR and no longer responsive to increases in extracellular dopamine [141]. These results suggest that neural circuits have been altered in SHR and that psychostimulant drugs affect SHR and WKY brains differently. This finding stresses the importance of studying animal models of ADHD as it shows that it cannot be assumed that drugs will have the same effect in children with ADHD and controls.

### Other animal models of ADHD

Several other animal models of ADHD have been proposed. These models were developed through genetic manipulation, exposure to toxins, rearing in social isolation, or interference with neurochemical systems. However, several do not satisfy the criteria for animal models of ADHD [45] and have therefore been excluded from the present review. These include the Naples high-excitability rat (NHE), WKHA rat, acallosal mouse, hyposexual rat, PCB-exposed rat, lead-exposed mouse, and rat reared in social isolation [45].

The reasons for exclusion are briefly as follows: NHE are hyperreactive in a novel environment, they are not hyperactive or impulsive in a familiar environment, and they have not been shown to be impaired in sustained attention [142,143]. WKHA rats are hyperactive but they are not impulsive [47,144-146]. The acallosal mouse becomes hyperactive over time and shows impaired acquisition of conditioned learning tasks [147]. However, impulsiveness decreases over time with repeated testing which is not characteristic of ADHD. Polychlorinated biphenyls (PCBs) administered either pre- or postnatally cause hyperactivity in rats but do not impair sustained attention [148-150]. Postnatal exposure of infant mice to lead causes ataxia and hyperactivity [151] but lead produces many other complications that would exclude a diagnosis of ADHD. Rat pups reared in social isolation display hyperactivity in a novel environment and increased errors of omission and perseveration [152]. However, these rats are not impulsive and are unimpaired in measures of task acquisition in the 5-choice serial reaction time (5-CSRT) test of sustained attention. In addition, children reared in social isolation would not be diagnosed as ADHD.

#### Coloboma mutant mouse

The SNAP-25 deficient mouse mutant coloboma (Cm/+) is of interest to ADHD because SNAP-25 polymorphisms have been associated with the disorder [153,154]. SNAP-25 regulates membrane trafficking and is involved in the release of all neurotransmitters as well as regulating translocation of proteins (e.g. NMDA receptor subunits) to the cell membrane. Altered expression of SNAP-25 will therefore have diffuse effects on neuronal function. The SNAP-25 deficient mouse mutant coloboma displays spontaneous hyperactivity [155] but lacks impulsiveness and has not been shown to have problems with sustained attention. Although the SNAP-25 deficient mouse does not model ADHD symptoms specifically, it may nevertheless serve as a useful model of non-specific brain dysfunction such as minimal brain disorder (MBD).

Depolarization-evoked (K^+^-evoked) release of glutamate from cortical synaptosomes is reduced in the coloboma mouse [156]. DRD2 expression is increased in the VTA and substantia nigra, suggesting increased inhibition of dopamine neuron firing rate [157]. Dopamine release and dopamine metabolites (DOPAC and HVA) are decreased in the striatum of coloboma mice which is consistent with decreased dopamine release and turnover [156,158] i.e. a hypofunctional dopaminergic system, similar to SHR. Striatal DRD1 and DRD2 expression is unaltered in the coloboma mouse [157]. Tyrosine hydroxylase expression is unaltered in VTA and substantia nigra whereas tyrosine hydroxylase and α_2A_-adrenoceptor expression is increased in the locus coeruleus of the coloboma mouse [157]. Noradrenergic function appears to be increased since experimental depletion of norepinephrine by DSP-4 reduces the hyperactivity of coloboma mice [159]. This suggests that motor activity in coloboma mice is caused by a hyperactive noradrenergic system but the hyperactivity is not completely abolished by depletion of norepinephrine, suggesting that additional factors contribute to the mutant phenotype [159], perhaps the imbalance between noradrenergic hyperfunction and dopamine hypofunction as suggested for SHR.

#### 6-OHDA-Lesioned Rat

Neonatal 6-OHDA-lesioned rats are not impulsive but they display hyperactivity and impaired learning in a spatial discrimination task, which improves after methylphenidate or d-amphetamine treatment [160-163]. Rat pups lesioned on postnatal day 1 displayed hyperactivity in adulthood [162]. They showed an initial decrease in spontaneous motor behaviour when placed in a novel environment, but after repeated testing their activity was increased relative to controls [162]. Hyperactivity was accompanied by decreased dopamine [162], increased DRD4 [164], and increased serotonin transporter binding in striatum but not cerebral cortex [165]. Hyperactivity was not altered by DAT1 inhibitors but was greatly reduced by DRD4 antagonists as well as inhibitors of SERT1 and NET1 [160,161,164,166]. These findings suggest that psychostimulants reduce hyperactivity of 6-OHDA lesioned rats not by inhibiting DAT1 but by inhibiting norepinephrine and serotonin transporters. Inhibition of NET1 would reduce dopamine uptake into noradrenergic terminals in several brain areas including prefrontal cortex and nucleus accumbens.

#### DAT-Knockout Mouse

DAT-knockout (DAT-KO) mice lack the gene that encodes DAT. These mice have been suggested as a model for ADHD because they are hyperactive in novel situations [167-169], have impaired extinction of responses in operant food reinforcement tasks [170]. They are also impaired in learning and memory tasks [168,169]. Impulsiveness has not been systematically investigated in DAT-KO mice. Although the absence of DAT is an extreme model of reduced midbrain DAT binding in adolescents with ADHD [36] it also contrasts with several studies that found increased DAT in striatum of ADHD children and adults [10,15,35]. The DAT-KO mouse nevertheless provides useful information concerning the neurobiological consequences of impaired DAT function.

Released dopamine is cleared at a slow rate giving rise to a 5-fold elevation of extracellular tonic dopamine in the striatum of DAT-KO mice i.e. a hyperdopaminergic state [171]. Electrically stimulated release of dopamine is decreased, suggesting that phasic release of dopamine is reduced i.e. the dopamine system is hypofunctional [171] similar to SHR and the coloboma mouse. However, unlike SHR, striatal DRD2 autoreceptors controlling dopamine synthesis are nonfunctional while DRD1 and DRD2 are downregulated by approximately 50% in the striatum of DAT-KO mice [171]. Hyperactivity in the DAT1 knock-out mouse might be the result of increased dopamine tone or decreased phasic dopamine release with consequently impaired activation of postsynaptic DRD1 required for LTP (and LTD) to produce changes in synaptic strength necessary for associative learning and reinforcement of appropriate behaviour.

Whereas specific inhibitors of NET1 or DAT1 did not affect DAT-KO hyperactivity, inhibitors of the SERT1 as well as drugs that activate the serotonergic system, such as serotonin receptor agonists and serotonin precursors, dramatically reduced hyperactivity [168]. DAT-KO mice provide convincing evidence that hyperactivity induced by high extracellular levels of dopamine can be reduced by enhancing serotonergic tone i.e. psychostimulants do not act via DAT1 to reduce hyperactivity in this model [168]. Interestingly, antagonists of the 5-HT_2A _receptor reverse the behavioural deficits of DAT-KO mice [172] and polymorphisms of the 5-HT_2A _receptor gene have been associated with ADHD [173,174]. While this model provides invaluable insight into possible mechanisms of psychostimulant action, the relevance of these findings to ADHD is not clear since serotonin reuptake inhibitors are of limited value in treating ADHD as one of the side effects of serotonin uptake inhibitors is stimulation of motor activity [38,168]. However, serotonin acts on a large number of receptor subtypes each with different spatial location and behavioural effects. Evidence obtained with the DAT-KO mouse suggests that specific antagonists of the 5-HT_2A _receptor deserve further investigation.

Interestingly, the DAT-knockdown (DAT-KD) mouse has been suggested as a model for obsessive compulsive disorder and Tourette's syndrome [175]. DAT knockdown, achieved by reducing DAT promotor strength, reduces adult DAT expression to 10% of wild-type levels and raises extracellular dopamine levels in the striatum to 170% of wild-type controls [175]. Hyperdopaminergic DAT-KD mice displayed excessive sequential stereotypy reflected as a complex serial pattern of grooming actions becoming more sequentially rigid and persistent. This type of behaviour is not characteristic of ADHD but may serve as a model for Tourette's and obsessive compulsive disorder [175]. Consistent with enhanced dopamine activation of DRD1 receptors being responsible for the excessively rigid serial pattern of instinctive grooming behaviour, DRD1 agonists produced similarly enhanced sequential stereotypy of syntactic grooming chains [175]. DAT-KD mice also tend to be hyperactive, to walk in perseverative straight paths, and to over-pursue certain incentive stimuli, consistent with obsessive compulsive disorder.

#### Poor 5-CSRT task performer

Rats that are selected for poor performance when trained in the 5-CSRT task provide a useful model of ADHD in that they are selected for deficient sustained attention, they show poor choice accuracy towards the end of testing sessions, and they demonstrate impulsiveness (premature responding) [176,177]. Methylphenidate treatment improved accuracy and reduced impulsiveness (at low doses) in poor performers [177]. Poor 5-CSRT task performers are not hyperactive and therefore may serve as a model of the inattentive subtype of ADHD.

In normal animals, response accuracy is adversely affected by activation of serotonin 5-HT_1A _receptors [178], while activation of 5-HT_2A _receptors increases the number of premature responses, suggesting that increased serotonin tone could be responsible for impulsivity of poor performers [179]. This is consistent with 5-HT_2A _receptor antagonists reversing the behavioural deficits of DAT-KO mice [172].

Evidence supports a role for dopamine in regulating the level of performance in the 5-CSRT task. In normal animals, d-amphetamine-stimulated release of dopamine in the nucleus accumbens caused a dose-dependent increase in premature responding [178]. Microinfusion of a DRD1 agonist into the medial prefrontal cortex selectively impaired the accuracy of attentional performance in high performers in the 5-CSRT task [180]. In contrast, microinfusion of the DRD1 agonist into the medial prefrontal cortex of poor performers enhanced the accuracy of attentional performance; a low dose increased the speed of making correct responses [180]. This finding once again emphasizes the need to study animal models of ADHD rather than normal animals in order to gain insight into the mechanisms that underlie the beneficial effects of drugs used to treat children with ADHD. Evidence suggests that the nervous system is altered in the 5-CSRT model of ADHD.

These results suggest that dopamine function is reduced in poor performers of the 5-CRST task and that 5-HT_2A _antagonists may be beneficial in the treatment of ADHD.

#### Anoxia in Neonatal Rat

Anoxia increases the risk of ADHD [181]. Neonatal anoxia caused a sequence of acute and persistent neurochemical changes in rat monoaminergic systems as well as transient hyperactivity and spatial memory impairment that persisted into adulthood [182-184]. Acute anoxia caused a transient decrease followed by an increase after 1 week in cerebellar norepinephrine levels [183]. At the same time, serotonin levels decreased while its metabolite, 5-hydroxyindoleacetic acid (5-HIAA), increased [183]. Striatal dopamine and metabolite concentrations decreased and then dopamine metabolites increased post ischaemia [183]. The increase in serotonin and dopamine metabolites persisted into adulthood, suggesting that dopamine turnover is increased. Tyrosine hydroxylase mRNA levels were increased in VTA and substantia nigra of perinatally asphyxiated rats suggesting increased dopamine synthesis consistent with increased turnover. However, DRD1 and DRD2 mRNA levels were increased in the striatum suggesting impaired release of dopamine [185]. These findings demonstrate the complex temporal sequence of compensatory changes that occur in monoaminergic systems following perinatal insult to the nervous system and implicate all three monoaminergic systems in spatial memory impairment.

### Insight provided by animal models of ADHD

One of the most important findings is the fact that animal models of both inattentive and hyperactive/impulsive subtypes of ADHD respond differently to psychomotor drugs when compared to controls suggesting that they have altered neurotransmitter systems in the brain. This emphasizes the need to study animal models of ADHD rather than normal animals in order to gain insight into the mechanisms that underlie the beneficial effects of drugs used to treat children with ADHD.

Neuroadaptations leading to psychostimulant drug addiction involve the same glutamate-dependent cellular mechanisms that enable learning and memory [87]. Similarly, neural mechanisms implicated in behavioural disturbances of animal models of ADHD are consistent with altered dopamine and/or norepinephrine mediated modulation of glutamate-dependent cellular mechanisms that enable learning and memory.

The various animal models of ADHD focus on different aspects of ADHD symptomatology and provide unique insights into ADHD neurobiology. They also emphasize the close interconnection between serotonergic, noradrenergic and dopaminergic systems. Changes in any one system can alter the function of the other monoaminergic systems and alter the underlying neural circuits that control behaviour.

There is convincing evidence of a dysfunctional dopaminergic system in several models of ADHD. The dopaminergic system appears to be hypofunctional in SHR, the coloboma mutant mouse, 6-OHDA lesioned rat, DAT-KO mouse (although extracellular hyperdopaminergia may also contribute to its behaviour) and poor performers in the 5-CSRT task. There is no convincing evidence to suggest that the underlying disturbance is primarily located in the serotonergic or other neurotransmitter system, although amelioration of ADHD symptoms may be partly mediated by drugs acting on the noradrenergic and serotonergic systems.

The dynamic developmental theory of ADHD [4] explains how behavioural changes associated with ADHD may result from deficient reinforcement and extinction processes as a result of a hypofunctional dopaminergic system and a steeper delay gradient in ADHD [4,186]. Impaired function of the mesolimbic dopamine system can cause ADHD symptoms of delay aversion, hyperactivity in a familiar environment, impulsiveness, deficient sustained attention, increased behavioural variability and failure to extinguish previously acquired behaviour [4]. Deficient mesocortical dopamine-mediated modulation of prefrontal cortical circuits can impair behavioural planning (executive function). Hypofunction of the nigrostriatal system would impair dopamine modulation of cortico-striato-thalamo-cortical circuits that control motor function giving rise to apparent developmental delay, clumsiness, and neurological "soft signs" [4].

The development of ADHD symptoms could be the result of inappropriately increased levels of dopamine at an early stage of development causing compensatory changes that subsequently give rise to hypofunctional dopamine neurons and impaired reinforcement/extinction mechanisms [4,139,186]. Children who have been exposed to elevated levels of brain dopamine prenatally as a result of mothers taking drugs of abuse, exhibit ADHD-like behaviour [187]. Exposure to drugs of abuse increases the extracellular dopamine concentration which reduces autoreceptor inhibition of VTA dopamine neurons and increases glutamate-driven activity of dopamine-containing neurons [187-190]. The consequences of increased extracellular dopamine is demonstrated in the DAT-KO mouse which loses the normal inhibitory effect of DRD2 on dopamine neuron firing rate in the VTA and substantia nigra, as well as dopamine release-regulating DRD2 function in the striatum [190]. The mechanism is suggested to involve increased AMPA receptor-mediated excitatory transmission and decreased inhibitory metabotropic glutamate receptor function in VTA dopamine neurons [187,188,191]. Increased activation by glutamate initially causes sensitization of VTA dopamine neurons with subsequent adaptations in the nucleus accumbens [191]. The increased glutamate drive is suggested to lead to pathophysiological consequences resulting from the high intracellular concentrations of Ca^2+ ^which gives rise to impaired function of VTA dopamine neurons and adaptation [191]. Similarly, ADHD symptoms may result from adaptation to initially increased extracellular dopamine in the VTA of the midbrain as a result of genetic and environmental effects at a very early stage of development giving rise to increased glutamate drive and subsequent loss of function of dopamine neurons.

The dynamic developmental theory of ADHD [4] explains how ADHD symptoms, including problems with sustained attention, can result from impaired dopamine function giving rise to a steeper delay gradient and poor stimulus control of behaviour when reinforcers are infrequent. Sustained attention is also controlled by noradrenergic projections from the locus coeruleus to the prefrontal cortex. There is considerable evidence to suggest that the noradrenergic system is poorly controlled by α_2_-autoreceptors in SHR, particularly at high norepinephrine release rates. This may be seen as hyperactivity of the noradrenergic system, especially when locus coeruleus neurons are stimulated in states of increased arousal. Increased release of norepinephrine from sympathetic nerve endings can give rise to the development of hypertension. Impaired regulation of norepinephrine release in the prefrontal cortex could give rise to ADHD-like symptoms.

It is interesting to note that although the various animal models have quite different origins, they have in common either increased or decreased tonic dopamine and/or decreased phasic release of dopamine. Some models also display poor regulation of locus coeruleus neurons and noradrenergic hyperactivity. These alterations may be primary or may reflect compensatory changes in response to more basic disturbances in neurotransmission, such as deficient SNAP-25 or impaired Ca^2+ ^signalling.

Future research should focus on determining the precise effects of psychostimulant drugs on the nervous system of animal models of ADHD. Psychostimulants affect animal models of ADHD and normal animals differently, suggesting that the nervous system of the ADHD model has undergone adaptive change which alters the effects of drugs used to treat ADHD. DAT-KO mice provide convincing evidence that psychostimulant drugs reduce hyperactivity by enhancing serotonin tone. Antagonists of the HT_2A_receptor are reported to reverse ADHD-like behaviour in some animal models. To test whether this is a general finding of relevance to ADHD, other animal models such as the SHR should be treated with HT_2A_antagonists to confirm whether their ADHD-like behaviour is similarly reversed. Once this is established, further testing to see if the beneficial effects of HT_2A _antagonists and psychostimulant drugs can be prevented by blockers of postsynaptic DRD1 receptors would be of interest, in order to test the dynamic developmental theory of ADHD [4] proposal that ADHD-like symptoms can be explained by deficient dopaminergic function and that psychostimulants enhance phasic dopamine release.

ADHD is a heterogeneous disorder, suggested to result from combinations of genetic and environmental factors. Animal models can mimic only certain aspects of the complex symptomatology of ADHD and at best provide feasible hypotheses regarding the underlying causes of specific aspects of ADHD behaviour. These hypotheses can then be tested in the clinic. Animal models can also be used to test potential drugs for the treatment of ADHD.

Future research on animal models of human disorders will undoubtedly promote a better understanding of the contribution of specific neurobiological factors to behavioural components like attention, reinforcement and extinction that seem to be important for understanding ADHD.
